# Characterization of *Prunus Necrotic Ringspot Virus* and *Cherry Virus* A Infecting Myrobalan Rootstock

**DOI:** 10.3390/v15081723

**Published:** 2023-08-11

**Authors:** Karima Ben Mansour, Petr Komínek, Marcela Komínková, Jana Brožová

**Affiliations:** 1 Ecology, Diagnostics and Genetic Resources of Agriculturally Important Viruses, Fungi and Phytoplasmas, Crop Research Institute, Drnovská 507, 161 06 Prague, Czech Republic; karina79@hotmail.fr (K.B.M.); kominkova@vurv.cz (M.K.); brozova@vurv.cz (J.B.); 2 Department of Plant Protection, Faculty of Agrobiology, Food and Natural Resources, Czech University of Life Sciences Prague, Kamýcká 129, 165 00 Prague, Czech Republic

**Keywords:** PNRSV, CVA, phylogenetic analysis, biological assay, symptoms

## Abstract

*Prunus necrotic ringspot virus* (PNRSV) and *cherry virus* A (CVA) are two viruses that mainly infect plants of the genus *Prunus*. Full-length sequences of these two viruses, collected in the Czech Republic from *Prunus cerasifera* plants, were obtained via HTS sequencing. Phylogenetic analyses based on the NJ method and Splitstree tools showed that the Czech PNRSV isolate (ON088600-ON088602) is a divergent isolate from other molecular groups, sharing less than 97% pairwise nucleotide identity with members of other groups. The Czech CVA isolate (ON088603) belonged to molecular subgroup III-2, clustered with isolates from non-cherry hosts, and shared the highest pairwise nucleotide identity (99.7%) with an isolate of Australian origin.

## 1. Introduction

Two viruses, *prunus necrotic ringspot virus* (PNRSV) and *cherry virus* A (CVA), are among the most common viruses infecting sour and sweet cherries in the Czech Republic [1].

PNRSV belongs to the genus *Ilarvirus* and infects *Prunus* spp. and ornamental plants [2]. It is a positive-sense single-stranded RNA virus. It has a segmented, tripartite genome, with RNA1 and RNA2 encoding two replicase proteins, P1 and P2, respectively, and RNA 3 encoding two other proteins: movement protein (MP) and coat protein (CP) [2]. Initially, PNRSV was divided into four different molecular groups (PV32, PV96, PE5, and CH30) [3]; then, another group appeared, the SW6 [4], and, recently, another (sixth) group was proposed, the PchMX-Azt [5]. However, compared to other molecular groups, most of the reported sequences are clustered into two major groups: PV32 and PV96 [6]. PNRSV is distributed worldwide. In the Czech Republic, it was first detected serologically in sour and sweet cherries [6]. PNRSV can be transmitted by pollen, which causes rapid virus spread in orchards [7], or by seed. These two natural modes of transmission have different efficiencies depending on the host plant species [8]. PNRSV is distributed also by infected plant-propagating material, such as budwood and rootstocks. The first symptoms of PNRSV appear one year after infection, called the acute or shock stage, but, later, plants become symptomless. However, previous studies have reported that some strains cause recurrent symptoms each year [9,10]. Infected plants show different symptoms depending on the PNRSV isolate, including mosaic, ringspot, chlorosis, leaf deformation, necrosis, shot holes, and drop-off. It also significantly affects fruit yield and quality. Infections with the virus may be latent or asymptomatic [2].

The second virus is *cherry virus* A (CVA), which belongs to the genus *Capillovirus* [11]. It is a positive single-stranded RNA with two ORFs; ORF1 encodes a replicase and coat protein, while ORF2 encodes a movement protein in a different frame [11]. The symptoms caused by this virus are considered latent or unknown because the virus is usually found in mixed infections, which complicates the association between this virus and specific disease symptoms [12]. This virus is distributed worldwide. It was first reported in 2010 in the Czech Republic in sweet and sour cherries [13]. The virus has also been found in non-cherry hosts, such as apricot, plum, peach, and Japanese apricot [14]. CVA has been divided into five molecular groups based on its RdRp [15] and six different molecular groups based on the complete genomes [14]. Recent studies have shown that CVA is clustered into seven phylogenetic groups [16]. CVA is transmitted via grafting; however, vector transmission has not yet been reported.

In the present work, these two viruses, PNRSV and CVA, were detected in a symptomatic myrobalan rootstock BN4Kr plant using high-throughput sequencing (HTS), and their complete genomes were assembled. To molecularly characterise these two Czech isolates, we screened them for recombination events using RDP4 and constructed a phylogenetic tree based on their complete coding regions. In addition, graft infections of different rootstocks were performed to determine the biological characteristics of these viruses and their corresponding symptomatology.

## 2. Materials and Methods

### 2.1. Plant Material and ELISA

In a previous study [17], several self-rooted plants of myrobalan BN4Kr (n = 55) were grown in a small open-field trial at the Crop Research Institute of Prague (CRI Prague). These plants were used to evaluate the field resistance of this rootstock (BN4Kr), together with six other rootstocks, to natural infection by plum pox virus (PPV) [17]. These plants remained PPV free throughout the four years of evaluation. Although no PPV was recorded on these plants, some ringspot symptoms were occasionally observed on them. In order to determine the causal agent behind this observation, a serological (ELISA) test using a commercially available antibody against PNRSV (Bioreba, Reinach, Switzerland) was performed to confirm the presence of this virus in these plants.

### 2.2. Sample Preparation and HTS

Total RNA was isolated from leaves of PNRSV ELISA-positive plant of myrobalan BN4Kr using a Spectrum Plant Total RNA Kit (Sigma-Aldrich, St. Louis, MO, USA). Ribosomal RNA was removed from the total RNA using the RiboMinus Plant Kit for RNA-Seq (Thermo Fisher Scientific, Waltham, MA, USA). The library for HTS was prepared using the TruSeq stranded mRNA Kit (Illumina, San Diego, CA, USA), with modifications to allow for processing of total RNA preparations according to the manufacturer’s instructions. Sequencing was performed on a MiSeq instrument (Illumina) at 2 × 150 nucleotides, resulting in 1,538,208 unique reads. Bioinformatic analysis was performed using Geneious Prime version 2020.2.4, as previously described [18], resulting in the assembly of the complete genomes of PNRSV and CVA.

### 2.3. Genome Characterization of PNRSV and CVA

The NCBI ORFfinder was used to predict the corresponding open reading frames (ORFs) from each sequence obtained (https://www.ncbi.nlm.nih.gov/orffinder, accessed on 22 May 2023). BioEdit 7.2.5 software was used to translate multiple nucleotide sequence alignments into their corresponding amino acid sequences [19]. The ExPASY ProtParam online application (https://web.expasy.org/protparam/, accessed on 31 May 2023) was used to predict the different characteristics of the PNRSV genome (molecular weight, aliphatic index, GRAVVY, GC content, total number of negatively charged residues, and total number of positively charged residues). Post-translational modifications (PTMs) were predicted using the ScanProsite server (https://www.expasy.org/resources/scanprosite, accessed on 5 June 2023).

### 2.4. Biological Assay and RT-PCR

Two myrobalan rootstocks (M29C and MRS 2/5) and apricot seedlings (M-VA-1) were inoculated with grafts from the PNRSV-infected BN4Kr plant. Ten plants from each rootstock were used for inoculation, and three uninfected plants were used as negative controls. The inoculated plants were grown in a screenhouse to avoid possible contamination by unwanted viral infections, and the inoculated and negative control plants were grown for five years, during which time leaf symptoms were assessed annually. The presence of the virus in the inoculated plants was further confirmed via both DAS-ELISA and RT-PCR using primers on the basis on the sequences obtained from HTS sequencing (Table S1).

### 2.5. Recombination Analysis

The genomes of PNRSV and CVA are tripartite and monopartite, respectively; so, four datasets were generated (Tables S2 and S3). Each dataset contained the Czech sequence—the subject of this study and all available sequences retrieved from NCBI. Thus, the first dataset contained RNA1 sequences of PNRSV (n = 41), the second dataset contained RNA2 sequences of PNRSV (n = 38), the third dataset contained RNA3 sequences of PNRSV (n = 92), and the fourth dataset contained sequences of the complete genome of CVA (n = 124).

The RNA1 and RNA2 datasets were aligned using the MAFFT online service [20] and trimmed to their respective coding regions using BioEdit version 7.2.5 [19]. To ensure that the alignment was in frame, the RNA3 and CVA datasets were prepared as concatenated ORFs aligned using the TranslatorX online server (http://translatorx.co.uk/, accessed on 23 May 2023) [21], using the encoded amino acids as a guide.

The RDP4 program was used to search for possible recombination events in these datasets using seven algorithms implemented in this program with default settings. The recombination event detected by at least three of these with a *p*-value < 10^−6^ was considered possible [22].

### 2.6. Phylogenetic and Sequence Demarcation Analyses

The three PNRSV datasets were shortlisted for phylogenetic analysis to include only the NCBI-retrieved isolates with complete genomes (all three RNAs). Recombinant isolates were also excluded. In total, there were 23 sequences for each RNA molecule. Seven additional reference sequences representing the six previously reported phylogenetic groups (PV32 = Y07568, PV96 = S78312, PE5 = L38823, CH30 = AF034994, SW6 = AF013287, and FJ546090-FJ546091 = PchMX-Azt) [3,4,5] were added to the RNA3 dataset (n = 30) (Table S3).

After removal of the recombinant sequences, the CVA dataset contained 105 sequences.

Phylogenetic trees were constructed based on the neighbor-joining (NJ) method using the MEGAX program [23], with a bootstrap set at 1000 replicates. A sequence demarcation tool (SDTv1.2) was used to determine pairwise nucleotide identity [24], and SplitsTree4.17.2 software was used to support the different clusters of these phylogenetic groups [25].

## 3. Results

### 3.1. Genome Organization of PNRSV and CVA

The myrobalan plant infected with PNRSV and CVA is deposited in the publicly accessible collection of plant viruses at the CRI Prague (collection acronym VURV-V, deposited under ref. no. VURV-V:46.2). The collection is accessible online via web hub www.microbes.cz (accessed on 31 July 2023). Virus isolates of PNRSV and CVA are named Ruzyne after the locality where they were found.

Full sequences resulting from HTS were deposited in GenBank under the following accession numbers: PNRSV (tripartite RNAs: ON088600, ON088601, ON088602) and CVA (ON088603).

The CVA genome has a GC content of 39.7% and consists of 7415 nt, containing two open reading frames of 7029 nt coding for both replicase and CP (2342 aa) and 1392 nt coding for MP (463 aa) in another frame.

The obtained PNRSV genome was found to consist of three RNAs, RNA1 with 3332 nt and RNA2 with 2591 nt, encoding P1 and P2 proteins of 1045 aa and 799 aa, respectively. The genome of RNA3 has 1944 nt, encoding 283 aa for MP and 224 aa for CP. The remaining characteristics of the RNAs mentioned in the Materials and Methods are listed in Table 1.

### 3.2. Recombination Analysis

Recombination analysis using the RDP4 programme showed that none of the Czech PNRSV segments were recombinant; however, seven isolates retrieved from NCBI (Q15R1N, TNpeach5, Che1, Che2, 13C257, 13C258, and 13C278) were recombinants. Two isolates obtained from NCBI showed recombination events only in their RNA1 (Q15R1N = KY883333, TNpeach5 = OL800569), two isolates showed recombination events in both RNA1 and RNA2 (Che1 = MH727235, MH727230, Che2 = MH727236, MH727231), and three isolates showed only one recombination event in their RNA3 (13C257 = MZ451054, 13C258 = MZ451055, and 13C278 = MZ451059) (Table S4).

The same result was obtained for the Czech CVA isolate, which had no recombination events. However, 19 CVA isolates from the NCBI database had at least one recombination event. About one-third had multiple recombination events. Most of the recombinant CVA isolates were obtained from cherries. The RDP4 programme showed that the Czech isolate (ON088603) was the putative major parent of the two NCBI isolates (LC422952, India, apricot) and (LC752551, Korea, sweet cherry) (Table S4).

### 3.3. Phylogenetic Analysis and Sequence Demarcation

**Figure 1:**
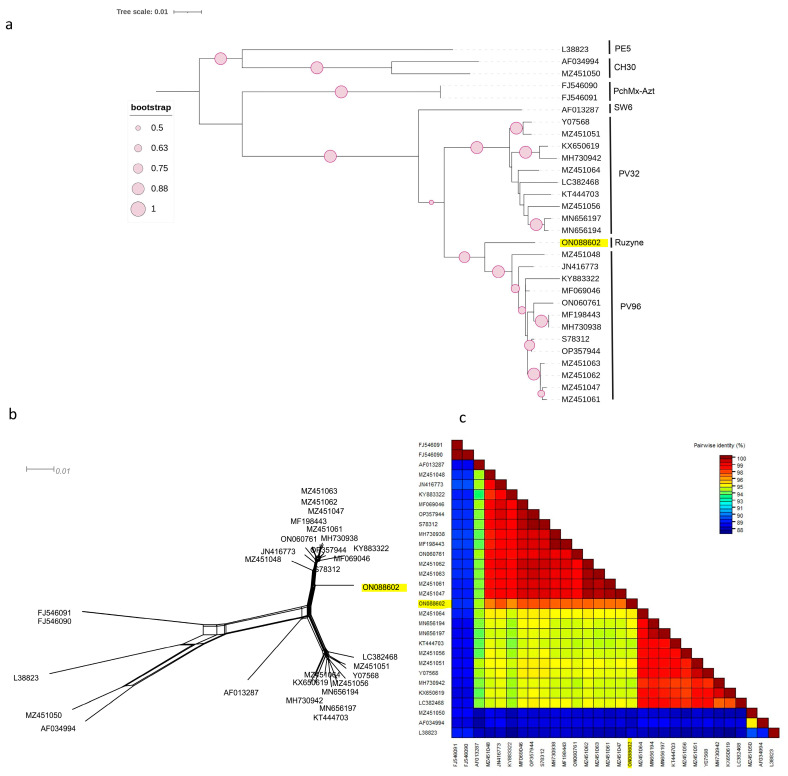
Characterisation of PNRSV based on the coding region of 30 RNA3 sequences using (NJ) mid-rooted phylogenetic tree (**a**), the phylogenetic network was examined using the Splitstree4.17.2. software (**b**), pairwise nucleotide identity analysis of different isolates using the SDT programme (**c**)**.**

Three individual phylogenetic trees were constructed from the complete coding regions of RNA1, RNA2, and RNA3 of PNRSV using the neighbour-joining method. The phylogeny was tested using the bootstrap method with 1000 replicates. The phylogenetic tree constructed on the basis of the complete coding region of RNA3 (Figure 1a) showed that the Czech PNRSV isolate (ON088602) clustered separately from the other five groups (PV32, PE5, CH30, SW6, and PchMX-Azt) in a sister clade to the PV96 phylogroup. One sequence (MZ451050) clustered within the CH30 phylogroup, whereas the remaining sequences clustered mainly within PV32 and PV96.

**Figure 2:**
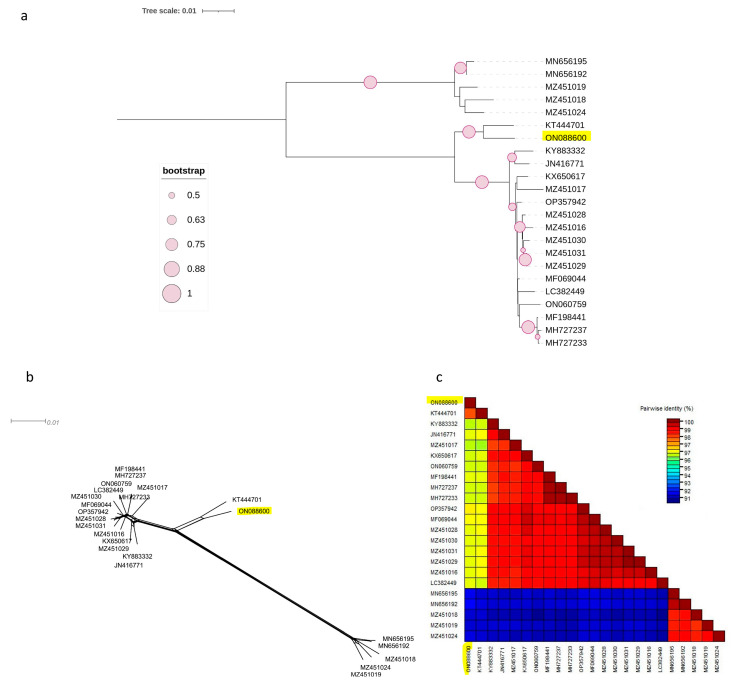
Characterisation of PNRSV based on the coding region of 23 RNA1 sequences using (NJ) mid-rooted phylogenetic tree (**a**), the phylogenetic network was examined using the Splitstree4.17.2. software (**b**), pairwise nucleotide identity analysis of different isolates using the SDT programme (**c**)**.**

**Figure 3:**
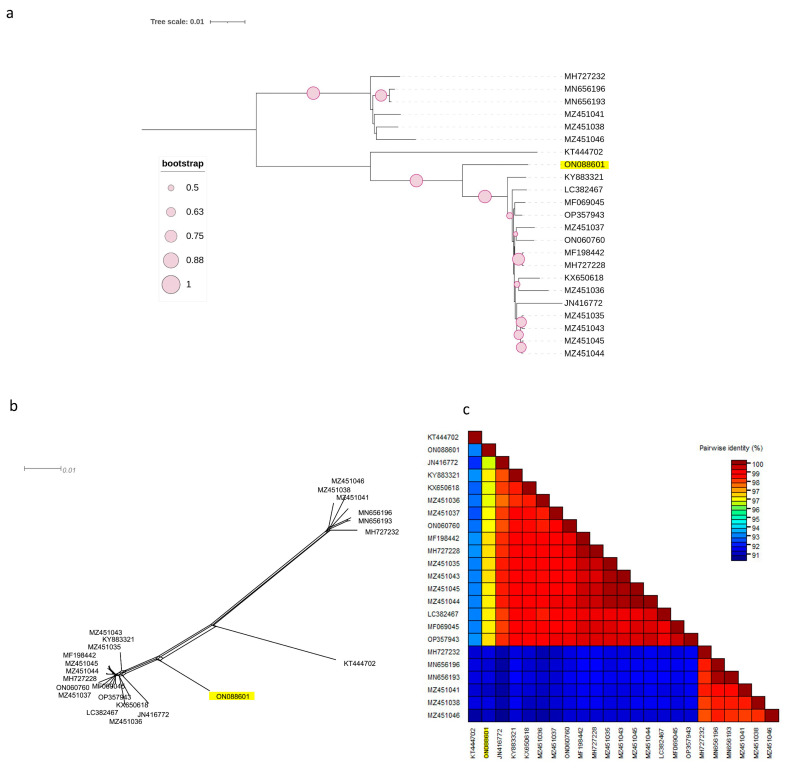
Characterisation of PNRSV based on the coding region of 23 RNA2 sequences using (NJ) mid-rooted phylogenetic tree (**a**), the phylogenetic network was examined using the Splitstree4.17.2. software (**b**), pairwise nucleotide identity analysis of different isolates using the SDT programme (**c**)**.**

The same observation was made for the other two phylogenetic trees based on the complete coding regions of RNA1 and RNA2 (Figure 2a and Figure 3a), in which the Czech isolate clustered separately from other molecular groups. Both phylogenetic trees had the same topology, with two phylogroups, I and II, in which clade II was divided into two subclades, II-1 and II-2. The Czech PNRSV isolate, the subject of this study, clustered within subclade II-1, separately from the other members.

This finding was further supported by the SplitsTree software v.4.17.2, which clearly showed that the Czech isolate was divergent from the other isolates (Figure 2b and Figure 3b). The SDT v2.1 programme showed that the pairwise nucleotide identities of the Czech isolate in RNA1 (Figure 2c) and RNA2 (Figure 3c) ranged between 91.7–96.7% and 91.2–96.9% with members of groups I and B, respectively. For RNA3 (Figure 1c), the Czech isolate shared pairwise nucleotide identities of 96.8–97.3%, 94.6–95.3%, and 87.7–94.4% with other PV96, PV32, and the remaining four molecular groups, respectively.

**Figure 4:**
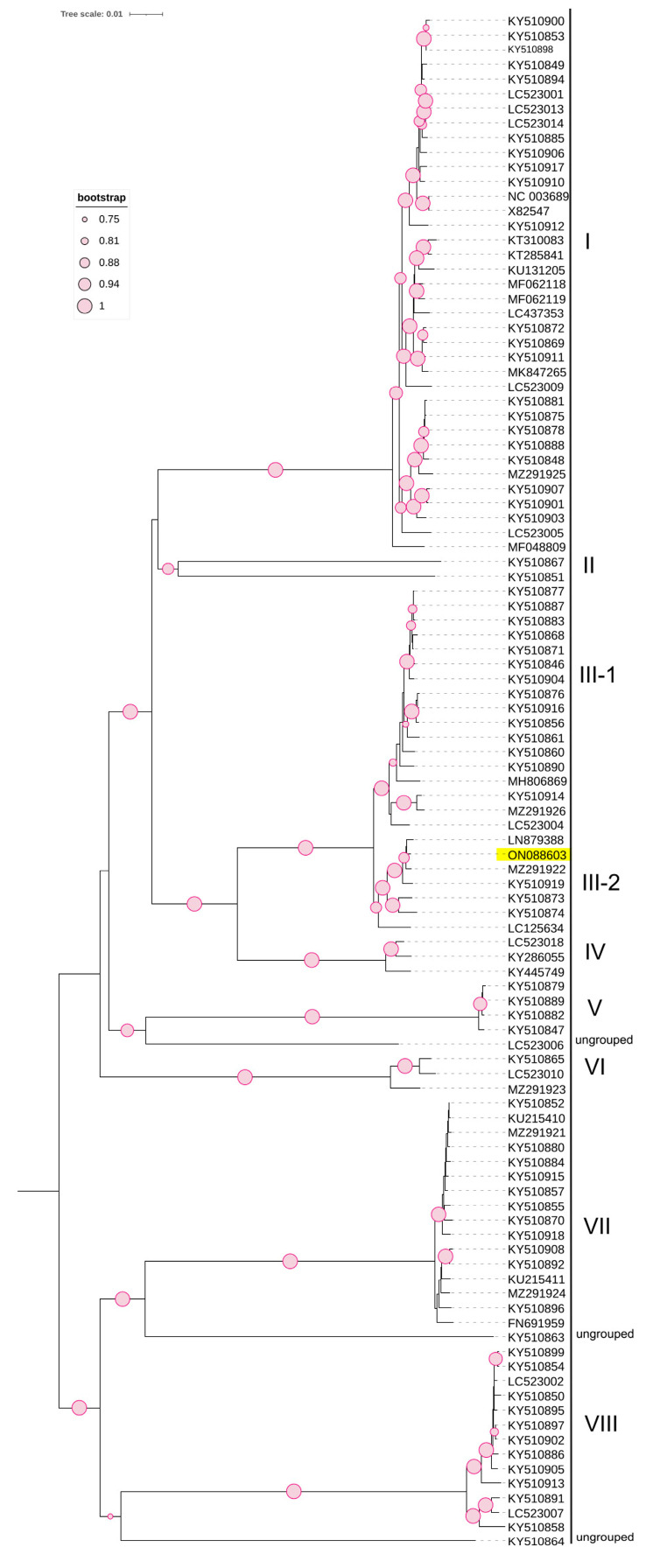
Phylogenetic tree based on the complete genome of nucleotide sequences of CVA constructed using the NJ method with 1000 bootstrap replicates.

Phylogenetic analysis based on the complete coding region of CVA (Figure 4), using 105 non-recombinant NCBI-retrieved sequences, revealed the presence of eight distinct molecular groups (I-VIII) and three ungrouped divergent isolates (LC523006, KY510863, and KY510864). The Czech CVA isolate (ON088603) clustered in subgroup III-2 with isolates from non-cherry hosts. The Czech CVA isolate was closely related to LN879388, an Australian isolate from the same host plant, *Prunus cerasifera* Ehrh., with a pairwise nucleotide similarity of 99.7%. These observed phylogenetic groups were further investigated using SplitsTree software v.4.17.2 (Figure S1), which confirmed this clustering, and SDT software v2.1 (Figure S2), which showed that pairwise nucleotide similarities between members of each group ranged from 97.9 to 100% and that nucleotide similarities between different groups ranged 81–87.1%. Notably, the lowest pairwise genetic similarities were observed for the divergent isolates (LC523006, KY510863, and KY510864), members of group II (KY510851 and KY510867), and group VI (MZ291923, LC523010, and KY510865), which ranged between 81.6% and 86.0% with members of other groups.

### 3.4. Variability of PNRSV Isolates

A comparison of the nucleotide sequences of the Czech PNRSV isolate (Ruzyne) with other sequences retrieved from NCBI, members of clades I and II-2, showed the presence of one SNP resulting in a non-synonymous amino acid substitution unique to the Czech isolate in coding regions of RNA1 at position 191. In addition to this unique SNP specific to the Czech isolate, it shared two SNPs with members of clade I, resulting in amino acid changes at positions 297 and 753, and 13 SNPs shared with members of clade II-2 (Table 2).

In RNA2 there were three SNPs unique to the Czech isolate compared to other isolates, and the three resulting substitutions were located at positions 35, 60, and 569. It shared three SNPs with members of clade I and 15 SNPs with members of clade II-2, resulting in amino acid substitutions in P2 (Table 2).

In MP, there was one unique amino acid change related to the Czech isolate, which was alanine, at position 49, compared to other isolates, all of which had isoleucine (Table 3).

In CP, the Czech isolate had an amino acid change (alanine) at position 135. In contrast, all other members of other groups had aspartic acid at this position (Table 3).

Prediction of potential PTMs revealed the presence of seven sites at positions 106, 433, 435, 436, 450, 753, and 959 in P1 and only one at position 135 in CP. These sites are putative targets for phosphorylation, N-glycosylation, and N-myristoylation.

### 3.5. Biological Assay

**Figure 5:**
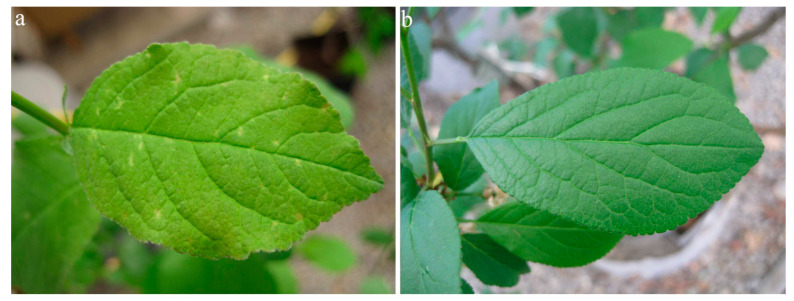
PNRSV symptoms on myrobalan MRS—mild spots on the leaf (**a**) compared to a healthy plant (**b**)**.**

**Figure 6:**
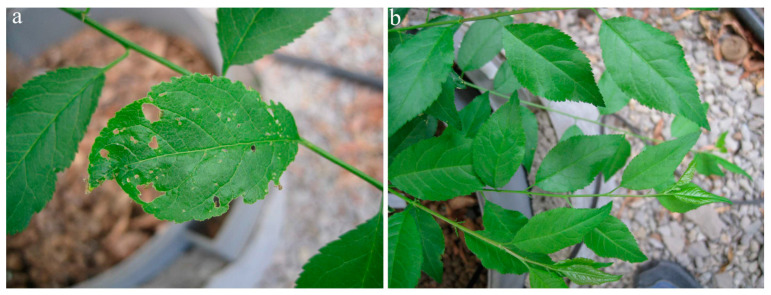
PNRSV symptoms on myrobalan M29C—tissue of ringspot had necrotised and dropped off (**a**) compared to a healthy plant (**b**).

In 2019, the first symptoms of infection were observed one year after graft inoculation. Virus-inoculated plants showed different systemic symptoms, ranging from mild spots in virus-inoculated plants of myrobalan MRS 2/5 (n = 10/10) (Figure 5) to necrotic spots in leaf tissue of plants of myrobalan M29C (n = 10/10) (Figure 6).

Symptoms appeared in spring and in about half of the number of shoots of the plant. They occurred in a part of the shoot, not in every leaf. Shoots grown during the summer showed no symptoms.

**Figure 7:**
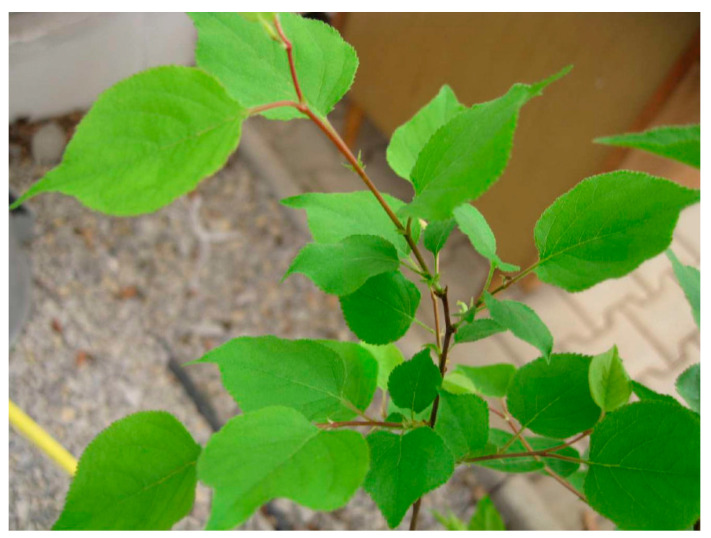
Apricot seedling inoculated with PNRSV, no symptoms.

Plants with mild symptoms or necrotic spots showed the same type of symptoms for the four years (2019–2022). However, in 2023, only mild mosaic symptoms were observed, and no necrotic ring spots were observed in both myrobalan genotypes. Apricot seedlings remained symptomless (0/10) during the five years of observation (Figure 7).

## 4. Discussion

Two of the most common viruses infecting plants of the genus *Prunus* occurring in the Czech Republic are CVA and PNRSV [1], where the latter virus is known to cause significant economic losses [26,27], especially since plants of the genus *Prunus* (sour cherry, sweet cherry, plum, apricot, peach, etc.) in the Czech Republic play an important role in the country’s economy.

Therefore, as a control measure against viruses infecting fruit trees, their propagating material is obliged to be tested for the presence of harmful viruses according to a Czech law (Act No. 219/2003 Coll. on the marketing of seeds and seedlings of cultivated plants and Decree No. 96/2018 Coll. on propagating plants and propagating material of fruit genera and species and their marketing). According to the abovementioned law, PNRSV is required to be tested in propagating material of sour cherry, sweet cherry, apricot, almond, peach, and plum.

Viruses infecting the genus *Prunus* have been monitored in Czech orchards [28], wild growing plants, including road trees of plums and myrobalans [29], and germplasm collections [1]. These studies confirmed that PNRSV and CVA are among the most common viruses infecting this genus in the Czech Republic [1].

In the current work, HTS analysis of symptomatic plants of BN4Kr myrobalan (*Prunus cerasifera* Ehrh.) revealed that these two viruses are present in a mixed infection, and the complete sequences of these viruses were obtained. The phylogenetic tree constructed on the basis of the complete coding region of RNA3 of PNRSV (Figure 1a) and using the NJ method showed that each reference isolate clustered in its appropriate molecular group, with most PNRSV isolates clustering in two groups, PV32 and PV96, in agreement with previous reports that PV32 and PV96 are the major groups. In comparison, the other four groups (PE5, CH30, SW6, and PchMx-Azt) were considered to be minor groups [30]. The Czech isolate clustered separately as a sister clade to the PV96 molecular group. Screening for SNPs leading to non-synonymous substitutions between the Czech isolate and members of other groups showed that it differed at one site in the MP and at another site in the CP. To test whether these differences might affect the virus life cycle, a search for putative targets of PTMs was performed using ScanProsite and revealed that one site (135) in the CP, which is unique solely to the Czech isolate, was a putative target for N-myristoylation. Although there has not been enough research into how myristoylation affects the life cycle of viruses, some studies have shown that this type of PTM can be involved in membrane targeting and binding [31] and can affect the structural and functional properties of proteins, such as stabilising the conformation (spatial structure) of the protein [32]. This analysis was extended to the P1 and P2 regions and showed that the Czech isolate shared more unique amino acids with subclade II-2 (n = 28) than with clade I (n = 5) and had four unique amino acids specific to it in both regions. The search for PTMs in P1 revealed that one site was a putative target for protein kinase C phosphorylation, four sites were putative targets for casein kinase II phosphorylation, and one site was a putative target for N-myristoylation, whereas, in P2, two sites were predicted to be targets for casein kinase II phosphorylation and protein kinase C phosphorylation, respectively. Although the difference between these molecular groups cannot be related to the severity of symptoms, as the sequences used for this analysis were retrieved from NCBI GenBank and have not previously been used for comparison between P1 and P2, a previous study in 2013 by Cui and co-authors [33] found that the C-terminal of RNA1 and the 2M region of RNA2 are required for severe virulence and high levels of viral accumulation. In our report, the highest number of sites found to be targeted by PTMs was in the replicase, where two sites, 753 and 959 in P1, corresponding to positions 2258 and 2875 in the C-terminal of RNA1, were targeted for protein kinase C phosphorylation and casein kinase II phosphorylation, respectively. This observation suggests that further studies are needed to determine the potential effects of these PTMs on the viral life cycle, particularly as some previous studies have suggested that phosphorylation of the replicase may affect the function of the protein. A study by Shapka et al. [34] showed that the phosphorylation of the *cucumber necrosis tombusvirus* (CNV) P33 replication protein renders it non-functional. Phosphorylation may also be involved in replication stability; for example, the phosphorylation of the 2a protein inhibits its interaction with the 1a protein of the *cucumber mosaic virus* (CMV) [35].

Two phylogenetic trees were constructed using the complete coding regions of RNA1 and RNA2 (Figure 1a and Figure 2a) and showed that the NCBI PNRSV isolates were divided into two major clades, I and II. Clade II was subdivided into two subclades, II-1, which included the Czech isolate, and subclade II-2. The result was similar to the previously reported phylogenetic trees constructed on the basis of the complete genome of RNA1 and RNA2 [36,37]. However, the difference between the previous studies and the present report is the observation of a new subclade II-1 containing the Czech PNRSV isolate. Kinoti and his co-authors, in 2017 [37], in their analysis of the intra-host genetic diversity of PNRSV, proposed a demarcation threshold for pairwise nucleotide similarities to distinguish different molecular groups (<97%). The pairwise nucleotide similarities (Figure 1b, Figure 2b and Figure 3b) between the Czech isolates and other members fell under this criterion, making the Czech PNRSV isolate a member of a new subclade, II-1, being a distant isolate from other molecular groups.

Graft testing was performed to determine the response of this divergent PNRSV isolate infection on rootstocks. Three rootstocks, Myrobalan M29C (*P. cerasifera*), MRS 2/5 (*P. cerasifera* × *P. spinosa*), and Apricot Seedling (M-VA-1), commonly used in the Czech Republic for their resistance to frost, root-knot nematodes, and their ability to improve fruit weight, were used for this purpose. The apricot seedling (M-VA-1) and myrobalan MRS 2/5 plants showed greater tolerance to the infection by being symptomless or showing mild spots, respectively. Myrobalan M29C plants showed severe symptoms with necrotic spots. Myrobalan M29C can be used as a biological indicator for the virus. Previous studies have established a chronology of the appearance of PNRSV symptoms, consisting of an acute or shock stage, one year after infection, after which, depending on the virus strain, the infected plant may either become symptomless or show recurrent symptoms annually [9,10]. In the case of this isolate, myrobalan M29C plants showed a mixture of these two patterns, starting with an acute stage, with annual recurrence of symptoms. However, after five years, the severe symptoms become mild. It is worth noting that the inoculum had CVA in addition to PNRSV, although CVA is thought to cause latent infection [12]; however, some synergistic effects affecting the severity of symptoms can be produced in the case of mixed infections [15].

The second virus molecularly characterised in this report is the CVA co-infecting the same plant, in which the phylogenetic tree constructed using all available and non-recombinant NCBI-retrieved CVA sequences showed the presence of eight molecular groups with no particular correlation to host plants. Previously, phylogenetic analysis of CVA resulted in five phylogroups based on the RdRp region [15], and six phylogroups were proposed based on the whole genome [14]. A later study showed that according to a phylogenetic tree belt based on the complete genome of 86 sequences, the CVA population could be divided into seven phylogroups, with five divergent isolates classified as ungrouped and likely to form other groups in the presence of more isolates [16], which is the case here—the eighth observed group was formed by one of these divergent isolates together with two newly available NCBI isolates. The Czech CVA isolate (ON088603) belongs to subgroup III-2 together with other isolates from non-cherry hosts. Recombination analysis revealed 35 recombination events in 19 NCBI isolates: 8 isolates from non-cherry hosts (*Prunus serrulata* Lindl., *Prunus mume* (Siebold) Siebold & Zucc., *Prunus cerasifera* Ehrh. and *Prunus armeniaca* L.) were inferred as parental sequences, with the Czech isolate (ON088603) identified as the parental sequence of two NCBI sequences.

Recombination can be a source of viral evolution, sequence diversity, and acquisition of new hosts [38]. An interesting observation was the presence of multiple recombination events in seven isolates, which usually indicates the presence of numerous viral isolates in the same plant, since recombination requires replication of two parental genomes in the same cell [39].

This work can contribute to the general knowledge of variability in PNRSV and CVA. Although phylogroups of the two viruses cannot be linked to the geographical origin of the virus isolates, some relationship with the Asian origin of the Czech sequences and, thus, the BN4Kr myrobalan genotype can be seen. International exchange of plant breeding material is the source of the introduction of new viruses or at least new phylogroups of existing viruses into new areas. Therefore, attention to phytosanitary measures in international trade and certification of plant-propagating material is still needed to control plant viruses.

## Figures and Tables

**Table 1:** Characteristic features of the Czech PNRSV genome.

	PNRSV			
	RNA1	RNA2	RNA3	
Accession number	ON088600	ON088601	ON088602	
Size (nt)	3332	2591	1944	
GC content (%)	45.4	41.7	46.8	
5′ and 3′ UTR (nt)	29 and 164	26 and 164	174 and 169	
Start-stop codon (position)	30–3167	27–2426	175–1026	1101–1775
Protein size amino acid (aa)	1045	799	283	224
ORFs encoded proteins	P1	P2	MP	CP
Theoretical Molecular Weight (kDa)	117.28	91.27	31.32	24.88
Aliphatic index	84.78	83.20	89.49	86.47
Theoretical isoelectric point (pI)	7.72	5.10	6.47	9.37
Grand average of hydropathicity (GRAVY)	−0.288	−0.234	−0.280	−0.277
Total number of negatively charged residues (Asp + Glu)	135	115	38	20
Total number of positively charged residues (Arg + Lys)	137	81	37	27

**Table 2:** List of polymorphic sites of RNA1 and RNA2 of Czech PNRSV isolate.

Gene	Position in Genome	Position in Protein	Czech Isolate		Clade I		Clade II-2	
			Codon	AA	Codon	AA	Codon	AA
RNA1	316	106	GCG	A	TCG	S	GCG	A
	573	191	CAG	E	CAT	D	CAT	D
	890	297	AGA	R	AGA	R	AAG	K
	1285	429	TCA	S	ATA	I	TCA	S
	1291	431	CCG	P	TCG	S	CCG	P
	1304	435	GTA	V	GAA	E	GTA	V
	1306	436	GCA	A	ACT	T	GCA	A
	1349/1351	450	AGT	S	AAT	N	AGT/AGC	S
	1378/1379	460	GTT	V	ACT/ATT/	I/T	GTT	V
	1409	470	GTT	V	GCT	A	GTT	V
	1983	661	CAG	Q	CAC	H	CAG	Q
	2258	753	AGG	R	AGG	R	AAG	K
	2875	959	TCA	S	ACA	T	TCA/TCT	S
	2902	968	GGT	G	AGT	S	GGT	G
	2995	999	TTG	L	ATG	M	CTG	L
	3100	1034	GCA	A	ACA	T	GCA	A
RNA2	25	9	TCA	S	ACA	T	TCA	S
	104	35	TTT	F	TCT	S	TCT	S
	142	48	ACT	T	GCT	A	ACT	T
	179	60	ATG	M	ACG	T	ACG	T
	373	125	ATG	M	CTG	L	ATG	M
	378	126	GAA	E	GAG	E	GAC	D
	388	130	TTC	F	GTC	V	TTC/TTT	F
	409	137	GTG	V	TTG	L	GTG	V
	451	151	ATG	M	ATG	M	GTG	V
	511	171	CCT	P	TTT	F	CCT	P
	523	175	ATC	I	GTC	V	ATC	I
	553	185	GTA	V	ATA	I	GTA	V
	558	186	GAT	D	GAA/GAG	E	GAT/GAC	D
	658	220	ATT	I	GTT	V	ATT	I
	664	222	TCG	S	GTG	V	TCG	S
	826	276	ATC	I	GTT/GTC	V	ATT	I
	1550	517	AAA	K	AGA	R	AAA	K
	1706	569	CTC	L	CCT	P	CCT	P
	2303	768	AAT	N	AAT	N	AGT	S
	2329	777	GCC	A	ACC	T	GCC	A
	2380	794	TGT	C	CGT	R	TGT	C

**Table 3:** List of polymorphic sites of MP and CP genes of Czech PNRSV isolate.

Gene	Position in Genome	Position in Protein	Czech Isolate		Other Six Molecular Groups	
			Codon	AA	Codon	AA
MP	145	49	GCC	A	ATC/ATT	I
CP	406	135	GCC	A	GAC	D

## Data Availability

Virus genomic sequences obtained in the present work have been deposited in the GenBank of the National Center for Biotechnology Information (NCBI) under accession numbers ON088600, ON088601, ON088602 and ON088603.

## Supplementary Materials

The following supporting information can be downloaded at: https://www.mdpi.com/article/10.3390/v15081723/s1, Figure S1: Phylogenetic networks examining 105 CVA complete genomes, created by SplitsTree v.4.17.2; Figure S2: Pairwise identity matrix of the complete sequences of CVA, created by SDT v1.2 software; Table S1: List of primers; Table S2: Accession numbers of NCBI-retrieved CVA sequences; Table S3: Accession numbers of NCBI-retrieved PNRSV sequences; Table S4: Recombination events detected in CVA and PNRSV.
