# Supplementary figures and images for: Characterization of Prunus Necrotic Ringspot Virus and Cherry Virus A Infecting Myrobalan Rootstock
Source: Viruses. 2023 Aug 11;15(8):1723. doi: 10.3390/v15081723 (PMC10459944; doi:10.3390/v15081723)

0.01

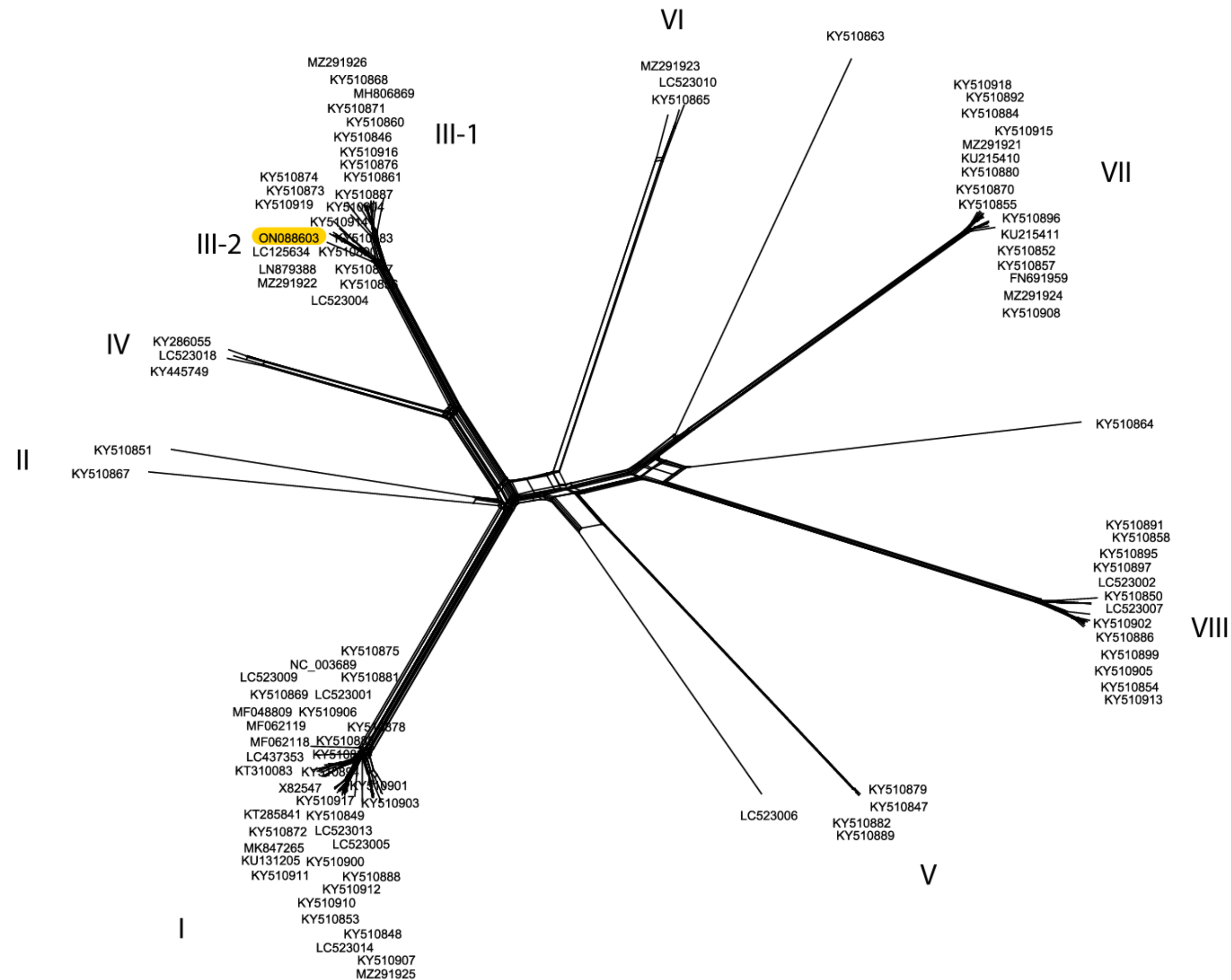

Figure S1. Phylogenetic networks examining 105 CVA complete genomes, created by SplitsTree v.4.17.2

Supplement: Supplementary file 1 [file viruses-15-01723-s001.zip › figure S-1.pdf]

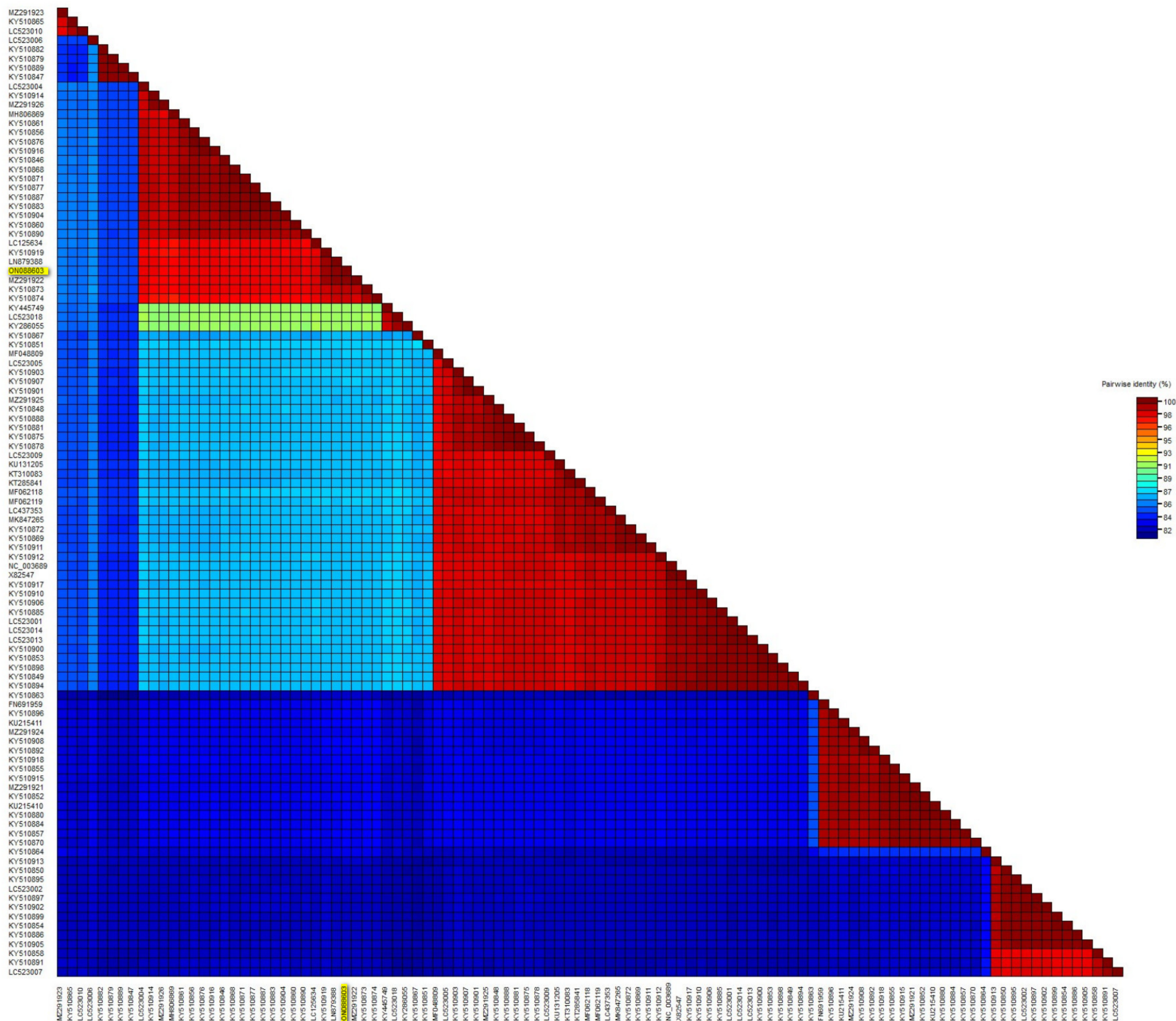

Figure S2. Pairwise identity matrix of the complete sequences of CVA, created by SDT v1.2 software

Supplement: Supplementary file 1 [file viruses-15-01723-s001.zip › Figure S2.pdf]
